# Growth of self-integrated atomic quantum wires and junctions of a Mott semiconductor

**DOI:** 10.1126/sciadv.abq5561

**Published:** 2023-05-03

**Authors:** Tomoya Asaba, Lang Peng, Takahiro Ono, Satoru Akutagawa, Ibuki Tanaka, Hinako Murayama, Shota Suetsugu, Aleksandar Razpopov, Yuichi Kasahara, Takahito Terashima, Yuhki Kohsaka, Takasada Shibauchi, Masatoshi Ichikawa, Roser Valentí, Shin-ichi Sasa, Yuji Matsuda

**Affiliations:** ^1^ Department of Physics, Kyoto University, Kyoto 606-8502, Japan.; ^2^ RIKEN Center for Emergent Matter Science, Wako, Saitama 351-0198, Japan.; ^3^ Institut für Theoretische Physik, Goethe-Universität, 60438 Frankfurt am Main, Germany.; ^4^ Department of Advanced Materials Science, University of Tokyo, Kashiwa, Chiba 277-8561, Japan.

## Abstract

Continued advances in quantum technologies rely on producing nanometer-scale wires. Although several state-of-the-art nanolithographic technologies and bottom-up synthesis processes have been used to engineer these wires, critical challenges remain in growing uniform atomic-scale crystalline wires and constructing their network structures. Here, we discover a simple method to fabricate atomic-scale wires with various arrangements, including stripes, X-junctions, Y-junctions, and nanorings. Single-crystalline atomic-scale wires of a Mott insulator, whose bandgap is comparable to those of wide-gap semiconductors, are spontaneously grown on graphite substrates by pulsed-laser deposition. These wires are one unit cell thick and have an exact width of two and four unit cells (1.4 and 2.8 nm) and lengths up to a few micrometers. We show that the nonequilibrium reaction-diffusion processes may play an essential role in atomic pattern formation. Our findings offer a previously unknown perspective on the nonequilibrium self-organization phenomena on an atomic scale, paving a unique way for the quantum architecture of nano-network.

## Teaser

A simple method to fabricate semiconducting atomic-scale networks is discovered, paving a unique path for a quantum architecture.

## INTRODUCTION

The fundamentals of device technology markedly change by the reduction of the dimensions. As the device size is reduced to the nanometer scale, the fabrication and integration of one-dimensional (1D) wire patterns become increasingly complicated and demanding. For the top-down approaches by using modern technologies with large-scale equipment, such as electron-beam and focused ion-beam lithography (*1*–*3*), fabricating patterns of nanowires with thickness and width less than ∼10 nm is technically challenging. On the other hand, bottom-up technologies by using self-assembly processes (*4*–*6*) suffer from controlling the uniformity of the wires. Moreover, in the bottom-up methods, the integration of nanowire arrays consists of two complicated steps: growing randomly oriented nanowires and aligning them into an array. Most of the recent growth of nanowires is based on the vapor-liquid-solid method (*7*–*9*). The assembly of the nanowires harnesses various processes such as Langmuir-Blodgett assembly (*10*, *11*). Therefore, the situation may call for a novel technology based on a fundamentally distinct concept that can fabricate uniform atomic-scale wires and engineer their nanopatterns.

Apart from the semiconductor technologies, atomic quantum wires of electron systems open a new route to realizing exotic phases of matter. For example, it has been proposed that in the proximity of a supercondcutor, atomic quantum wires can host Majorana fermions, which can provide an approach to constructing a fault-tolerant quantum computer (*12*). Also, the quantum wires consisting of correlated electrons provide a platform for the physics of 1D non–Fermi liquid described by such as Tomonaga-Luttinger model, in which spin-charge separation plays an important role (*13*). Furthermore, when the electron correlations are strong enough in the half-filled band, Mott insulators that can be seen as 1D quantum magnets are realized. An exotic example of such a system is an Ising magnet forming a trijunction (Y-junction), which has an exactly solvable ground state hosting Majorana fermions (*14*). Therefore, the fabrication of quantum wire arrays and junctions in correlated systems has the potential to substantially affect the field of condensed matter physics.

Here, we demonstrate that uniform and long single-crystalline wires of β-RuCl_3_ in an atomic scale are reliably fabricated by a simple deposition technique. Furthermore, we manufactured several characteristic patterns pivotal for realizing quantum nanocircuits, including atomically smooth junctions and nanorings. β-RuCl_3_ is an interesting material because it is a Mott insulator in which electron-electron interaction opens a gap, while at the same time its bandgap is comparable to those of wide-gap semiconductors. The formation of these nanowire patterns takes place as a part of the thin-film growth, but the growth process is essentially different from the conventional ones. In the present process, the formation and integration of nanowires are achieved simultaneously, making this method unique and attractive for applications. Furthermore, we obtain multiple pieces of evidence that the observed atomic-scale wire patterns emerge as a result of a characteristic self-organization process. We will discuss that the nonequilibrium reaction-diffusion processes may play an important role for the uniformly aligned patterns. We will further point out a possible atomic-scale Turing mechanism (*15*), which has been discussed for macroscopic pattern formations in chemistry (*16*) and biology (*17*–*19*).

## RESULTS

**Fig. 1.**
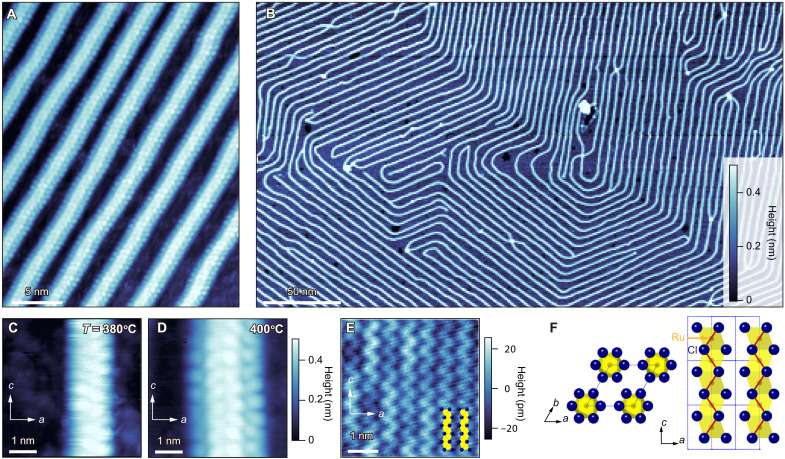
Topographic images of β-RuCl_3_ atomic-scale wires grown on HOPG surfaces. (**A**) Topographic images highlighting atomic structures of the β-RuCl_3_ wires consisting of four β-RuCl_3_ single-crystalline chains. Periodic white spots represent chlorine atoms. The deposition temperature is 400°C. The color scale is shared by (A) and (B). The images are taken at 2 V and 30 pA. (**B**) A topographic image of β-RuCl_3_ on HOPG taken at 3 V and 20 pA. Bright lines represent single-crystalline β-RuCl_3_ wires with four–unit cell width and dark blue areas represent *a*-Ru-Cl, an amorphous material consisting of Ru and Cl. The deposition temperature is 400°C. (**C** and **D**) Topographic images highlighting atomic structures of the β-RuCl_3_ wires consisting of two (C) and four (D) β-RuCl_3_ single-crystalline chains. The deposition temperatures are 380°C (C) and 400°C (D). The color scale is shared by (C) and (D). The images are taken at 2 V and 50 pA for (C), and 2 V and 30 pA for (D). (**E**) A topographic image of 2D monolayer β-RuCl_3_ taken at 3 V and 50 pA. Zigzag chains of chlorine atoms are arranged in parallel. (**F**) Crystal structure of β-RuCl_3_ viewing from directions normal to *ab*- (left) and *ac*- (right) planes. The blue dashed lines denote the unit cell. In the right panel, the monolayer crystal structure is shown. Zigzag red lines correspond to the zigzag chains of Cl atoms in (E).

We evaporated high-quality RuCl_3_ on the surfaces of a highly oriented pyrolytic graphite (HOPG) by the pulse-laser-deposition (PLD) method (see Materials and Methods). Figure 1 (A to E) shows the scanning tunneling microscope (STM) topographic images of the growth results. Shown in Fig. 1A is an atomic-resolution image of a sample grown at the deposition temperatures of 400°C. We find that the surface is covered by a unique pattern of wires (bright lines), which are ordered and almost evenly spaced. Each wire consists of periodically spaced atoms, demonstrating the single-crystalline structure. Figure 1B displays a typical large-scale picture of the growth, revealing that the wires grow in some preferred directions with being connected smoothly at the corners. The direction is parallel to the zigzag direction of the carbon honeycomb lattice of the substrate (orange arrow in fig. S1B). Reflecting the crystal structure of HOPG, the growth direction of wires is oriented 60° away from each other. The regions between the atomic wires (dark blue areas in Fig. 1, A and B) are filled with a thin material. Because no periodic lattice structure is seen (fig. S1A), the thin material is in an amorphous form consisting of Ru and Cl, denoted as *a*-Ru-Cl.

To identify the material forming the atomic-scale wires, we extend the deposition time to grow 2D monolayer and thicker films. As shown by the STM image in Fig. 1E, we can obtain single-crystalline 2D monolayer films. No wires are formed on the monolayer film, enabling continuous layer-by-layer growth (*20*). On the basis of the combination of the characteristic zigzag chains and x-ray diffraction pattern of the thicker films (fig. S4), we identify the crystal as β-RuCl_3_. The zigzag chains originate from the topmost Cl atoms as highlighted by the red lines in Fig. 1F. The periodicity along the chain corresponds to the lattice constant of β-RuCl_3_, further corroborating that the chain is β-RuCl_3_. The atomic wires shown in Fig. 1A have the same periodicity as the β-RuCl_3_ chain along the wire direction, and their widths coincide with double (Fig. 1C) and quadruple (Fig. 1D) β-RuCl_3_ chains. The same zigzag structure is resolved only in the inner chains of four–unit cell–wide wires. The apparent difference between the inner and outer chains may be caused by scanning an edge (the outer chain in this case) using a tip with finite curvature or may reflect the difference in the electronic states of the inner and outer chains. As discussed later[Fig F6], the height of the wire is ∼0.5 nm, which is consistent with the monolayer thickness of β-RuCl_3_. The height of *a*-Ru-Cl is typically less than 0.2 nm, much smaller than β-RuCl_3_.

**Fig. 2.**
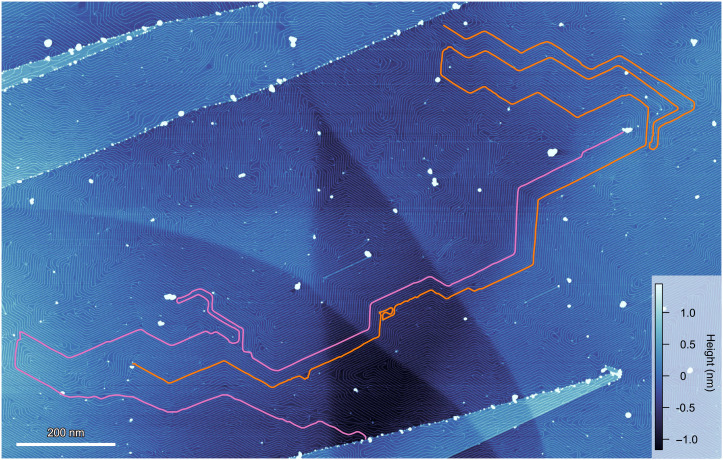
Topographic image of β-RuCl_3_ atomic wires extending over a few micrometers. The orange and magenta lines are overlaid on atomic wires of β-RuCl_3_ with a four-unit-cell width (∼2.8 nm). Their lengths are longer than 3 μm. The high clusters are objects adhering to the surface probably during the growth process. The topographic image was taken at 3 V and 20 pA.

A salient feature of the wire is its length. As demonstrated in Fig. 2, the length is as long as more than 3 μm (a length-width ratio is more than 1000), and both the width and height of the wire are quite uniform over its entire length. This length is substantially longer than most of the nanowires with a few atomic widths reported so far, which are typically 10 to 100 nm lengths. To our knowledge, the presently observed patterns of uniform 1D atomic wires with mesoscipic length scale are unique and unprecedented.

**Fig. 3.**
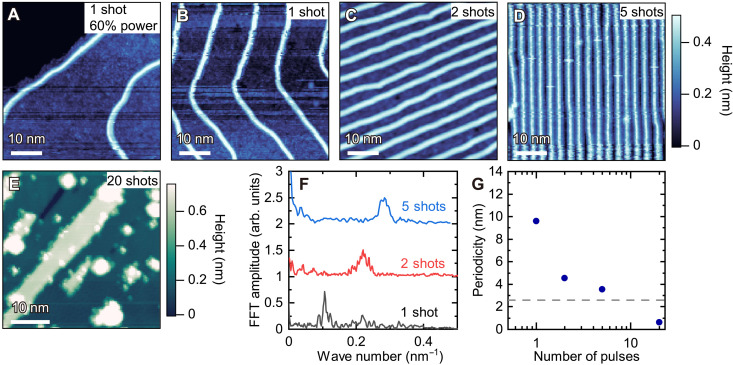
Stripe patterns of β-RuCl_3_ atomic-scale wires. (**A** to **D**) Topographic images of β-RuCl_3_ wires with four–unit cell width grown at 400°C. By changing the deposition time of the laser from one to five shots, the wire distance can be tuned from much longer than 10 nm (A) to shorter than 2 nm (D). The power of the laser pulse is further attenuated to 60% for (A). The color scale is shared by (A) to (D). (**E**) A topographic image of a β-RuCl_3_ monolayer thin film grown by a further increase of the deposition time to 20 shots. Green and white regions correspond to mono- and double-layer thick β-RuCl_3_, respectively. No 1D wire pattern is observed. The setpoint conditions are 20 pA and 3 V [(A), (B), and (E)] and 30 pA and 3 V [(C) and (D)]. (**F**) Line profiles of fast Fourier transform (FFT) images in the direction of peaks corresponding to the wire repetition. The curves are vertically shifted for clarity. (**G**) The periodicity (the inverse of the wave number) is plotted as a function of the number of pulses. The dashed gray line indicates the width of the four-chain wire. The data point for the 20 shots represents the lateral lattice constant of monolayer β-RuCl_3_.

On the basis of the above results, we conclude that the atomic wires consist of two or four β-RuCl_3_ single-crystalline chains growing in the [001]-direction directly on the HOPG surfaces. At the deposition temperature of 400°C, the wires always consist of quadruple chains of β-RuCl_3_ (Fig. 1D), while the width is reduced to double chains at the lower deposition temperature of 380°C (Fig. 1C). Hereafter, “four- (two-) chain rule” denotes the mechanism by which four (two) chains bundle together to form a single wire. The detailed mechanism of the four (two)-chain rule is not apparent, and exploring its quantum-mechanical origin deserves future studies. However, this rule is crucial for building a model, as discussed later. We note that the *c*-axis lattice constant of β-RuCl_3_ does not match the lattice constants and their integer multiple of the carbon honeycomb lattice. Therefore, the epitaxial coupling with HOPG is not essential for the 1D wire formation, further supported by the curved 1D wires shown in Fig. 3A.

As shown in Fig. 3 (A to D), the spacing between the 1D wires is widely tunable ranging from lengths longer than 10 nm (Fig. 3A) to less than 2 nm (Fig. 3D), by changing the deposition time. The longer deposition time results in the formation of the 2D monolayer β-RuCl_3_ thin film (Fig. 3E). A more quantitative analysis using the fast Fourier transform is shown in Fig. 3F. The periodicity of the wire, the inverse of the wave number at the peak position in Fig. 3F, monotonically decreases as increasing the deposition time (Fig. 3G). For the five shots, the periodicity is 3.5 nm, which is merely above the width of wires (∼2.8 nm), indicating that the wire separation is less than 1 nm.

**Fig. 4.**
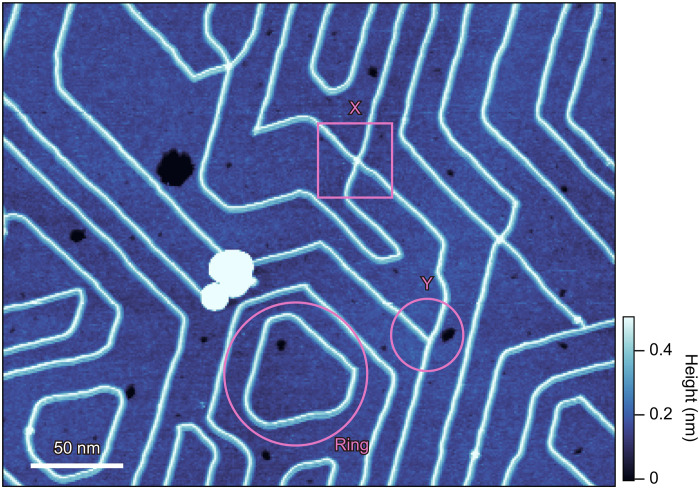
Spontaneous formation of junctions and rings. A topographic image taken at 20 pA and 3 V shows X-, Y-junctions, and rings of four–unit cell–wide β-RuCl_3_ wire.

Another few intriguing patterns found in addition to the stripe patterns are X-, Y-junctions as well as nanorings composed of atomic-scale wires, as indicated in Fig. 4. The height of the nanoring and Y-junction is uniformly one unit cell. These results demonstrate the fabrication of atomically smooth junctions and rings without introducing defects and clusters, which are crucially important for nanocircuits.

**Fig. 5.**
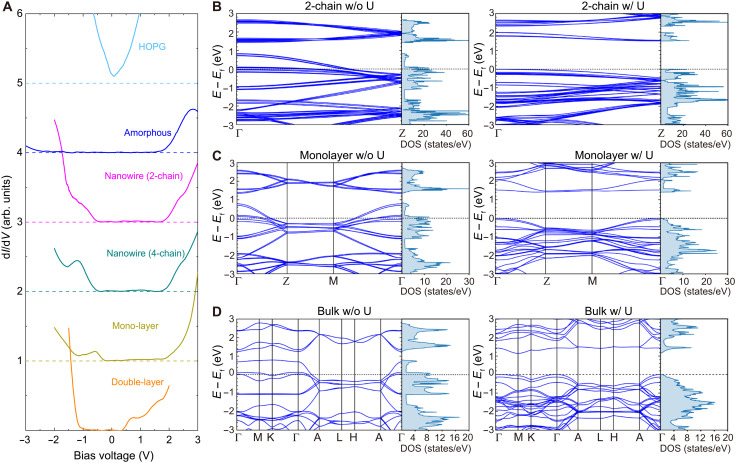
Electronic structures of β-RuCl_3_ atomic-scale wires. (**A**) d*I*/d*V* spectra of β-RuCl_3_. The setpoint conditions are 2 V and 100 pA (double-layer), 3 V and 100 pA (mono-layer and 2-chain nanowire), 3 V and 50 pA (four-chain nanowire and amorphous), and 1 V and 100 pA (HOPG). The spectra of four-chain nanowire and amorphous are doubled so that the setpoint current is equivalent to the others. The curves are vertically shifted for clarity, and each dashed line represents the base line. We did not specify a chain in a wire due to the piezo creep being relatively large at 78 K. (**B** to **D**) The calculated electronic band structure of two-chain wire, monolayer, and bulk β-RuCl_3_, respectively. The left (right) columns show the calculations without (with) electronic correlations. DOS, density of states.

To reveal the electronic structures, we measured the differential tunneling conductance (d*I*/d*V*) spectra (Fig. 5A), which are proportional to the local density of states, on wires with two– and four–unit cell width. For the comparison, spectra of 2D β-RuCl_3_ with mono- and double-layer thicknesses (Fig. 3E), *a*-Ru-Cl, and HOPG are also shown. In contrast to semimetallic HOPG with a linear dispersion, clear gap structures are seen in all β-RuCl_3_, indicating the semiconducting or insulating electronic structures. To unveil the origin of the energy gap, we performed systematic band calculations of a two-chain wire, a monolayer, and bulk β-RuCl_3_, considering electron correlations and spin-orbit interactions (Fig. 5, B to D). All the cases are metal with a finite density of states at the Fermi energy unless the electron correlation is included, whereas when it is turned on, an energy gap opens at the Fermi energy for all cases, indicating that β-RuCl_3_ is a Mott insulator.

Considering Fig. 3 (A to E), the formation of the nanowire array appears to be part of the thin-film growth. However, it should be emphasized that this crystal growth process is essentially different from any of the growth processes previously known: island, layer-by-layer, and their combination (*20*–*22*). Note that the spontaneous formation of distinctive stripe patterns has been reported in macroscopic and mesoscopic scales in various chemical and biological systems. Two contrasting approaches have been advocated to explain the stripe pattern formation in the previous systems. One is an equilibrium process, in which the periodic spatial organization appears as a result of the static potentials consisting of competition between attractive and repulsive interactions with two different length scales or purely repulsive isotropic interactions (*23*–*25*). The other is to view the 1D stripe pattern as the result of a dissipative structure that exhibits complex nonequilibrium reaction-diffusion.

**Fig. 6. F6:**
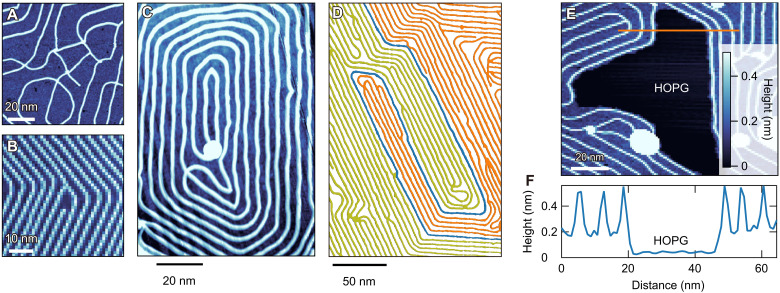
Topographic images exemplifying spatial patterns formed by reaction-diffusion processes. (**A**) Multiple crossings of atomic wires of β-RuCl_3_ with four–unit cell width observed in a region with very low wire density. The color scale is shared by (A) to (C) and (E). (**B**) Termination of a wire with four–unit cell width accompanied by a Y-shaped junction. (**C**) Winding spiral pattern. (**D**) Collision of growing waves of atomic wire colored in yellow and orange, forming a spiral pattern. The boundary of the waves is colored in blue. The raw image is shown in fig. S2. (**E**) A depleted region of *a*-Ru-Cl, where the HOPG surface appears. Atomic-scale wire propagation terminates at the boundary of this region. (**F**) Height profile along the orange line indicated in (E). The setpoint conditions are 20 pA and 3 V (A), 30 pA and 2 V (B), 30 pA and 2 V (C), 30 pA and 3 V (D), and 20 pA and 3 V (F).

To elucidate the mechanism of the present pattern formation, noteworthy about the observed patterns is the emergence of several distinct characteristic features displayed in Fig. 5 (A to E), in addition to the stripe patterns. It should be stressed that from these peculiar patterns, static interactions are highly unlikely to be the driving force of the atomic-wire array formation. As shown in Fig. 6A, when the distance between the wires becomes longer, the stripe patterns disappear, but the wire crossings still remain. These results contradict the pattern formation driven by static interactions, whether repulsive or attractive, because crossings typically induce energy loss if static interactions drive the pattern formation. If attraction-driven, it is more stable to condense into a 2D phase separation structure, i.e., crystalline domains. In the present case, such a crystallized domain has not been observed. For the repulsion-driven case, the uniformly dispersed pattern is more stable, which contradicts the present case with dense and sparse regimes coexisting, as shown in Fig. 6A. Thus, the possibility of the static interactions can be ruled out as the origin of the atomic-wire patterns.

We point out that nonequilibrium reaction-diffusion processes may play an essential role in forming the observed peculiar atomic-wire patterns. In reaction-diffusion systems, the crossings are allowed to form (*26*). In addition, as depicted in Fig. 6B, when the number of wires changes, the Y-junction accompanied by an edge dislocation appears. For a stripe pattern, the number of lanes’ discordance is typically resolved in two ways. One is to narrow the distance between the lanes. The other one is to form transiently or stationary emerging Y-junctions, edges of lanes, and shrinking lanes, which we observed in Fig. 6B. Such a pattern is a typical fingerprint for the reaction-diffusion process (*15*, *27*).

Moreover, the presence of the phase singularity, a unique feature in reaction-diffusion systems (*28*), is demonstrated by the spiral patterns in Fig. 6 (C and D). Figure 6C shows a typical winding spiral pattern (see also fig. S6). The pattern in Fig. 6D is created as a result of two waves, one colored yellow and the other orange, propagating from different directions and colliding with each other. We emphasize that these spiral patterns cannot be generated in the equilibrium systems, providing strong evidence for the nonequilibrium process (*26*, *28*) (see also the Supplementary Materials). Furthermore, when the deposition temperature is lowered to 380°C, the HOPG surface with no *a*-Ru-Cl appears as shown by the black area in Fig. 6E. The wires do not propagate through a depletion region by themselves without accompanying *a*-Ru-Cl regions nearby, which is confirmed by the height profile (Fig. 6F) along the orange line in Fig. 6E. The absence of the propagation of wires to the depletion region is consistent with the reaction-diffusion picture because it suggests that the production of atomic-scale wires regulates the concentration of the *a*-Ru-Cl and vice versa.

We note that the STM is too slow to capture the dynamic process of the thin-film growth. Therefore, even though these STM results support the reaction-diffusion process as the origin of the atomic-wire pattern formation, measurements that can directly detect dynamical processes in atomic-scale are strongly desired to fully understand the growth mechanism.

## DISCUSSION

Assuming that the reaction-diffusion process is the origin of the pattern formation of atomic wires, it is tempting to speculate the stripe pattern to be a manifestation of Turing instability (*15*). This instability is the most prominent mechanism in many different classes of self-organization processes, leading to the spontaneous emergence of spatially periodic patterns (*15*). In the following, we discuss the possibility that the observed patterns are associated with the Turing instability.

Turing patterns occur when the diffusion coefficient of an inhibitor is substantially larger than that of an activator; local activation and lateral inhibition destabilize uniform stationary states. After the first experimental realization of Turing patterns around 1990 (*29*, *30*), 40 years after Turing’s prediction, the Turing mechanism has been recognized as a prominent driving force that can induce the chemical (*16*) and biological (*17*–*19*) pattern formations. However, despite its applicability to various systems with a wide length scale ranging from more than centimeters to submicrometers (*31*), the atomic-scale formation of the Turing pattern down to a few nanometers has been seldom examined. Very recently, striped wrinkle patterns of the 2D bismuth atoms on NbSe_2_ have been discussed in the analogy of the Turing pattern by associating the atomic displacements with activator and inhibitor (*32*). However, further evidence is required to clarify whether these patterns appear because of the nonequilibrium self-organization process because similar patterns can be induced even by an equilibrium process stemming from the static interactions (*23*–*25*). In addition, the present finding of the atomic-scale wire fabrication is distinct from these wrinkle patterns on 2D continuous films, which is essentially a surface reconstruction phenomenon. Thus, the mechanism that underlies diffusion-driven instability on the atomic scale, where quantum mechanics is essentially important, can be fundamentally different from that on the larger scale. Moreover, it is an open question whether the large differences in diffusion rates of reacting species can be satisfied on the atomic scale.

**Fig. 7.**
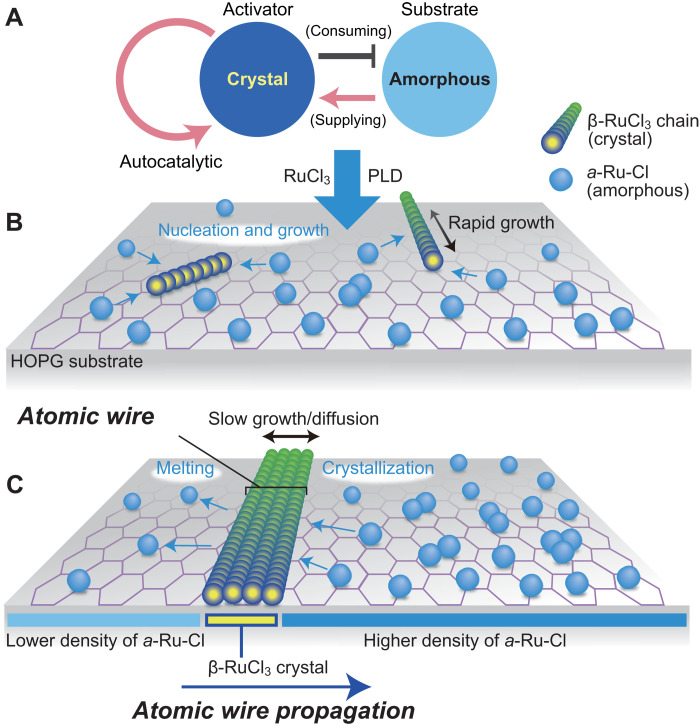
Schematic diagrams of the atomic-wire formation by Turing mechanism. (**A**) Activator-depleted substrate scheme. Depletion of the substrate acts as an inhibitor in the conventional activator-inhibitor system in the Turing mechanism. (**B** and **C**) Crystal growth and diffusion process of β-RuCl_3_. The chemical reaction process occurs on both sides of the 1D wires to form and decompose β-RuCl_3_, but the reaction is activated more frequently on the side with a higher concentration of *a*-Ru-Cl. The atomic wires propagate toward the direction with a higher concentration of *a*-Ru-Cl. This process describes the reaction-diffusion origin of the pattern formation.

Despite that the Turing mechanism in atomic scale is largely unexplored, we point out that some of the observed patterns appear to be consistent with this instability. Let us deduce an underlying model that describes the present system following the Turing instability. The emergence of the depletion areas shown in Fig. 6E suggests the activator-depleted substrate scheme (*33*), where the “substrate” denotes the chemically reacting substance. As schematically shown in Fig. 7A, β-RuCl_3_ crystal is an activator that enhances nucleation growth and consumes the substrate *a*-Ru-Cl, and the depletion of the substrate acts as an inhibitor in the conventional activator-inhibitor model for the Turing process. We illustrate the crystal growth and diffusion process in Fig. 6 (B and C). The crystal growth of 1D β-RuCl_3_ wires proceeds along the chain ([001]) direction because of the quasi-1D crystal structure of β-RuCl_3_.

Because the reaction between the wire and amorphous is activated more frequently on the side with a higher concentration of amorphous, the wires propagate toward the direction with a higher concentration of *a*-Ru-Cl, as illustrated in Fig. 7C. In a typical crystal growth, the crystal reacts with *a*-Ru-Cl, resulting in the lateral growth (layer-by-layer growth). However, in the present case, the atomic wires do not laterally grow but rather propagate toward the direction with a higher concentration of *a*-Ru-Cl due to the four-chain rule. The diffusion rate of the β-RuCl_3_ crystal is substantially slower than that of *a*-Ru-Cl because of the substantial difference between particle hopping rates in them. The diffusion rate is proportional to $\sqrt{N}$, where *N* is the size of the substance. Because the typical volume of β-RuCl_3_ wire is more than 100 times larger than that of *a*-Ru-Cl, the diffusion rate of β-RuCl_3_ is expected to be more than 10 times smaller than *a*-Ru-Cl. Note that the volume of a-Ru-Cl is ∼0.2 nm by 0.2 nm by 0.2 nm, while that of the wire is 2 nm by 0.6 nm by *L* nm, where *L* is the length of the wire. *L* is typically longer than 100 nm in the present case. This suffices the necessary condition for the Turing instability that the diffusion coefficient of the inhibitor is substantially larger than that of the activator. As shown in fig. S5, a reaction-diffusion model describing the activator-substrate system exhibits a stripe pattern through the Turing instability (*15*) (see the Supplementary Materials). Also, we note that while the uniform stripe pattern appears when the density of atomic wires is high (as shown in Fig. 3, C and D), the pattern is not periodic when the density of atomic wires is low (Figs. 4 and 6A). We point out that this nonperiodic pattern does not rule out the possibility of Turing mechanism as the origin of the uniform pattern at high-density (see the Supplementary Materials).

The present method of growing uniform atomic wires also has the potential to be applied to a wide variety of systems and opens up avenues for the future of nanowire technology. The robust and flexible growth of wires on the atomic terraces has great advantages for the applications. Applications include the use of atomic patterns as lithographic masks and the use of the nanowires themselves. We also point out that the present results are important for the basic science. As shown in Fig. 5, β-RuCl_3_ is a Mott insulator with *J*_eff_ = 1/2, in which the electron correlation is predominant for the low-energy electronic states. We note that the gap formation due to Peierls instability is excluded because no dimerization is observed in the STM images. In this case, the present β-RuCl_3_ wires provide a platform for the physics of pure 1D quantum magnets described by such as Tomonaga-Luttinger liquid and Haldane gap systems (*34*). Also, it has been known that another polymorphic form α-RuCl_3_ with 2D honeycomb lattice is a candidate material of a Kitaev quantum spin liquid hosting Majorana fermions (*35*–*37*), which can provide an approach to constructing a fault-tolerant quantum computer (*12*). Combined with α-RuCl_3_, the atomic-scale wires and junctions of β-RuCl_3_ might be useful for quantum circuits that use Majorana fermions. Furthermore, nanowire fabrication could be realized by the same mechanism in materials with 1D structure similar to β-RuCl_3_. For instance, transition metal trihalides with the same or similar quasi-1D crystal structure as β-RuCl_3_ have recently been focused on, and our method may be applied to this family (*38*). Other exciting candidates with quasi-1D crystal structures include transition metal chalcogenides such as WTe_2_ (*39*) and *M*_6_*X*_6_ (*40*).

The fabrication of nanowires and junctions has the potential to dramatically increase the integration of electronic circuits. The present 1D crystalline atomic wires and their networks provide fascinating physical playgrounds of nonequilibrium self-organization phenomena in atomic-scale, exotic electronic states and quantum technologies.

## MATERIALS AND METHODS

### Sample growth

Atomic-scale wires and thin films of β-RuCl_3_ were grown on the surfaces of HOPG by the PLD method. To obtain a fresh surface, the HOPG was cleaved, followed by annealing at 400°C for 15 min. The pressure of the PLD chamber was kept at about 10^−5^ Pa during the deposition. An 81-mJ pulsed yttrium-aluminum-garnet laser operating at a wavelength of 1064 nm was used to vaporize a solid target of high purity α-RuCl_3_ single crystals, which were synthesized by the chemical vapor transport method. To grow the films with atomic-layer thickness, a 16- to 18-ns pulsed laser was used, and the laser power was tuned by using the attenuator. We find that the β phase of RuCl_3_ can be grown at HOPG temperature between 350° and 400°C in a high vacuum. The growth of β-RuCl_3_ was confirmed by x-ray diffraction analysis of the thicker films grown on the same deposition conditions. The growth condition was controlled by changing the number of pulses, laser power, and HOPG temperature. After the growth, the films were transferred by a portable vacuum chamber to the STM chamber to image topographies and measure the local electronic properties.

### STM measurements

The STM experiments were performed with a custom-made Unisoku ultrahigh vacuum STM at 78 K. The STM tips were electrochemically etched tungsten wires cleaned by electron-beam heating and conditioned on Ag(111) surfaces evaporated on Si(111) surfaces. The bias voltage was applied to the sample. All topographic images were taken in the constant-current mode. The differential conductance was measured with the standard lock-in technique at 973 Hz with a modulation voltage of 20 mV.

### Density functional theory calculations

All calculations are performed on the experimentally obtained crystal structure taken from Ref (*41*). The structure is in the space group 185 *P*6_3_*cm* with lattice parameters *a* = *b* = 6.120 Å and *c* = 5.658 Å, where the Ru-Ru chains propagate in the c direction. The Ru-Ru distance within the chain is 2.829 Å and there is no dimerization. This is in agreement with the presented STM images in this work which do not observe any dimerization either.

The experimental structure has been additionally compared with the fully relaxed structure obtained within density functional theory (DFT) via the VASP simulation package (*42*) version 6.3.0. As exchange-correlation functional, we considered the generalized gradient approximation (GGA) (*43*) where we included the Coulomb correction via the Dudarev (*44*) GGA + U scheme with an effective Coulomb repulsion *U*_eff_ = 3.7 eV. The simulations have been performed with a planewave cutoff of 600 eV for the expansion of the basis set, and on a 8×8×6 k-mesh. The relaxation is performed including spin-orbit effects and van der Waals corrections via the DFT + D2 method of Grimme (*45*), using a ferromagnetic state on the Ru sites, until the forces for each atom in all directions decrease down to 10^−3^ eV/Å. The comparison shows that the relative distances between the atoms deviate by a small amount, approximately 0.02 Å, with respect to the experimentally reported distances, and therefore, we continue with the experimentally reported structure.

To calculate the electronic properties of the experimental system, we use the full potential local orbital (*46*) package version 21.00–61 and the GGA (*43*) where we include the Coulomb correction for the strongly localized Ru 4d electrons via the GGA + U approximation using the atomic limit method (*47*). We apply a Coulomb correction of *U* = 4.1 eV and *J* = 0.37 eV, which corresponds to approximately *U*_eff_ = 3.7 eV. This value is slightly higher than reported constrained random-phase approximation values for a related Ru-system, the α-RuCl_3_ compound (*48*). Because β-RuCl_3_ consists of quasi 1D chains, we expect that the screening effects will be slightly reduced and thus the effective Coulomb repulsion should be slightly larger. All calculations are spin-polarized with ferromagnetically aligned magnetic moments on the Ru sites. Because of the heavy Ru atoms, we perform fully relativistic calculations with a spin quantization axis in the *a*-direction.

The bulk calculations are performed on a 12×12×12 k-grid in the primitive unit cell and density convergence criterion 1 × 10^−6^. For the monolayer setting, we performed a slab calculations in the *a-c* plane and considered void space in the *b* direction on a 10×1×10 k-grid. In the two-chain arrangement, we use void space in the *a* and *b* direction and 1×1×10 k-grid. The monolayer and two-chain structure are both converged up to density accuracy of 1 × 10^−4^.
