# Supplementary material for: Growth of self-integrated atomic quantum wires and junctions of a Mott semiconductor
Source: Sci Adv. 2023 May 3;9(18):eabq5561. doi: 10.1126/sciadv.abq5561 (PMC10156112; doi:10.1126/sciadv.abq5561)
Supplement: Supplementary file 1 — Supplementary Text Figs. S1 to S6 [file sciadv.abq5561_sm.pdf]

Science Advances
AAAS

Supplementary Materials for

Growth of self-integrated atomic quantum wires and junctions of a Mott semiconductor

Tomoya Asaba *et al.*

Corresponding author: Tomoya Asaba, asaba.tomoya.4t@kyoto-u.ac.jp;
Yuji Matsuda, matsuda@scphys.kyoto-u.ac.jp

Sci. Adv. **9**, eabq5561 (2023)
DOI: 10.1126/sciadv.abq5561

The PDF file includes:

Supplementary Text
Figs. S1 to S6

Other Supplementary Material for this manuscript includes the following:

Movies S1 and S2

REACTION-DIFFUSION MODEL

To explain the stripe pattern of β-$RuCl_3$, we consider a reaction-diffusion model describing the activator-substrate system illustrated in Fig. 4a. Let a and s be the concentration fields of activator (crystalline β-$RuCl_3$) and substrate (amorphous a-Ru-Cl), respectively. The important reaction is that a unit constituting the amorphous becomes a part of the crystal at the rate proportional to the concentration of the crystal. This auto-catalytic reaction is suppressed by the depletion of the substrate when its diffusion constant D_s is much larger than activator's diffusion D_a. The mechanism leads to the Turing instability of a spatially homogeneous state. As one example, the equations for a and s are written as

$$\partial_t a = sa - a - 1 + D_a(\partial_x^2 + \partial_y^2)a, \tag{S1}$$

$$\partial_t s = -sa + 2 + D_s(\partial_x^2 + \partial_y^2)s, \tag{S2}$$

where we set $D_a = 1$ and $D_s = 20$ to satisfy the condition $D_s \gg D_a$. The last condition seems reasonable because the particle hopping rate in the crystalline β-$RuCl_3$ is much smaller than that in amorphous a-Ru-Cl. Here, the isotropic diffusion is assumed, for simplicity. It should be noted that the form of Eqs. (S1) and (S2) was introduced by Turing.

By a standard method, it is easily confirmed that the spatially homogeneous state $(a, s) = (1, 2)$ is unstable against periodic perturbations. We then numerically solve Eqs. (S1) and (S2) with the boundary conditions $\boldsymbol{n}\boldsymbol{\nabla}a = \boldsymbol{n}\boldsymbol{\nabla}s = 0$, where $\boldsymbol{n}$ is the unit vector perpendicular to boundaries. We choose an initial condition randomly near the homogeneous state. Furthermore, in solving the equations, we impose the following additional rule: if the concentration a is zero and the increasing rate of a is negative, a remains zero. This special rule was also mentioned in the paper by Turing.

An example of the resulting stationary pattern is shown in Extended Data Fig 6, which shows an irregular stripe pattern. Also, in Supplementary Movie 2, we show the simulated nucleation and propagation of wires. Since thin film growth usually occurs from singularities such as clusters of impurities or steps, the graphite substrate steps are considered to be the nucleus. Considering this, in this simulation, we use Eqs. (S1) and (S2) as well, but assume that the initial concentration of a is dense along the left edge of the figure (indicated by warm color). The movie shows that the ordered wire pattern is spontaneously formed.

Although this simulation based on simple assumptions should be scrutinized more closely,

it captures an essential feature of the observed patterns.

SPIRAL FORMATION OF STRIPES

Figure 3d shows a spiral of stripes, which is quite unique even in reaction-diffusion systems. Let us consider a model equation that exhibits such a pattern. First, when the Turing instability occurs, a plane-wave perturbation with wavenumber vector $\boldsymbol{k}$ grows at the rate $\lambda(\boldsymbol{k})$. The simplest example of $\lambda(\boldsymbol{k})$ is given by

$$\lambda(\boldsymbol{k}) = r - (|\boldsymbol{k}|^2 - k_c^2)^2, \tag{S3}$$

where $r = 0$ corresponds to the onset of instability and k_c is the critical wavenumber at $r = 0$. The exponential growth due to the instability is saturated by a non-linear effect, while its detailed mechanism depends on the system under study. Here, we assume that the activator is described by a two-component field associated with a crystal structure. Let W be the complex field corresponding to the two-component field. The equation for W is then written as

$$\partial_t W = (1 - (\partial_x^2 + \partial_y^2 + 1)^2)W - (1 + ic)|W|^2 W, \tag{S4}$$

where we have set $(r, k_c) = (1, 1)$ in Eq. (S3), and the last term represents non-linear saturation. Equation (S4) with $c = 0$ is called the Swift-Hohenberg equation, which describes periodic pattern formations in many systems including the Rayleigh-Bénard convection. The important property of the Swift-Hohenberg equation is that W evolves with decreasing a free energy (or a potential function). It has been known that spirals are never observed in such potential systems. Now, for the case $c \neq 0$, the system does not possess a potential, and thus spirals may be formed. Supplementary Movie 1 displays an example of the time evolution of the real part of W for $c = 1$.

Supplementary Movie 1 An example of the time evolution of the real part of W for $c = 1$. See section **Spiral formation of stripes** for details.

Supplementary Movie 2 An example of the time evolution of a in Eqs. (S1) and (S2). See section **Reaction-diffusion model** for details.

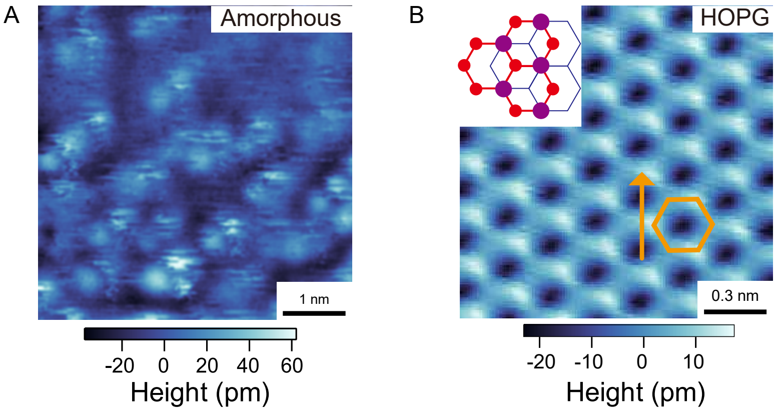

FIG. S1. **Topographic images of amorphous and HOPG. A,** Typical atomic-scale image of a-Ru-Cl, which fills the areas between the wires (dark blue areas in Fig. 1A and Fig. 1B taken at 3 V and 20 pA. No periodic structure is seen. **B,** Atomic-scale image of HOPG surface. The orange hexagon and arrow indicate the carbon honeycomb lattice and the chain direction of β-$RuCl_3$. . The setpoint conditions are 1 V and 100 pA.

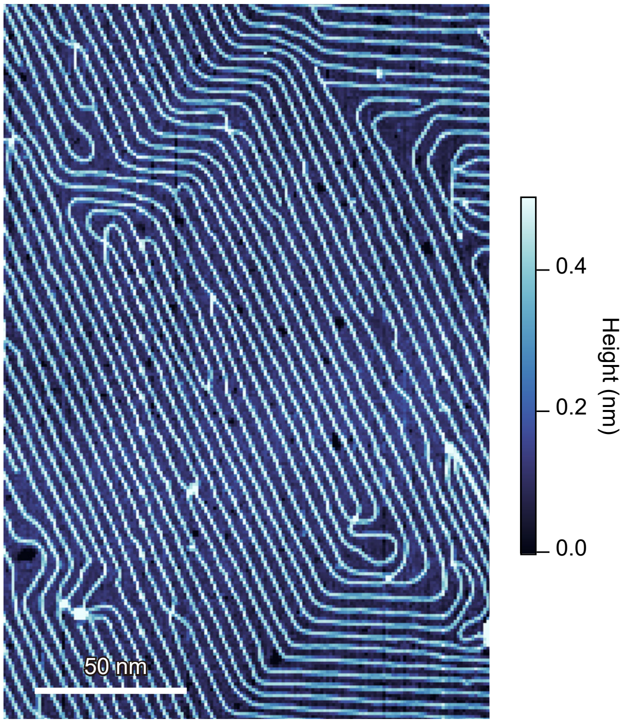

FIG. S2. **The raw topographic image of the spiral pattern shown in Fig. 6D.**

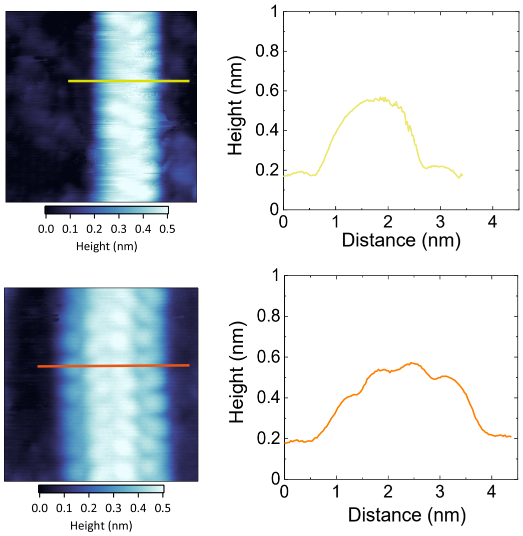

FIG. S3. **Atomic wires with different widths.** (left) Topographic images of atomic wires with two- (top) and four- (bottom) unit-cell widths, which are grown at 380 °C and 400 °C, respectively. (right) The height profiles along the lines shown in the left panels. The base line is set to the HOPG surface.

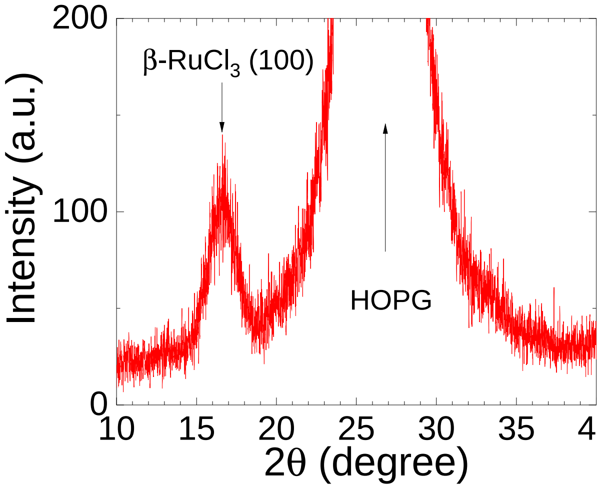

FIG. S4. **X-ray diffraction pattern of a β-RuCl$_3$ thin film.** A peak at 2θ=16.7° corresponds to the (100) plane of β-RuCl$_3$. The thickness of the film is less than 100 nm.

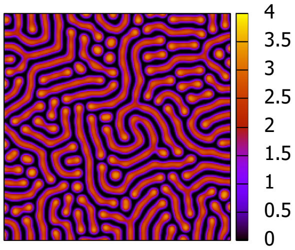

FIG. S5. **Simulated Turing pattern.** See SI for details. The simulated spiral pattern is also shown in Supplementary Movie 1.

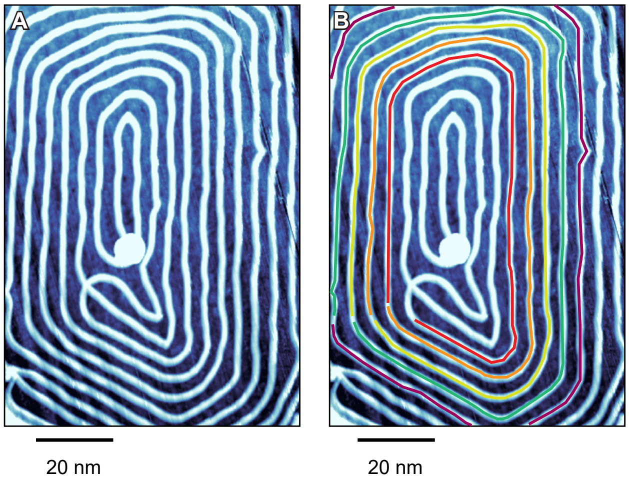

FIG. S6. **A spiral pattern with multiple windings. A,** The same image as shown in Fig. 6C. **B,** The same image as **A** with a different color for each lap of the spiral to make the spiral pattern easier to see.
